# *Chlamydophila psittaci* pneumonia followed by lower gastrointestinal ischemic necrosis: a case report

**DOI:** 10.3389/fmed.2024.1394897

**Published:** 2025-01-08

**Authors:** Shifeng Shao, Jun Liu, Zhenbing Wu, Shasha Wu

**Affiliations:** Medical Center of Trauma and War Injury, Daping Hospital, Army Medical University, State Key Laboratory of Trauma and Chemical Poisoning, Research Institute of Surgery, Chongqing, China

**Keywords:** psittacosis, parrot fever, chlamydial pneumonia, metagenomics next generation sequencing (mNGS), abdominal surgery, critical care

## Abstract

**Background:**

Psittacosis, also known as parrot fever, is an uncommon infectious disease caused by *Chlamydophila psittaci* (C. psittaci). While *C. psittaci* infections are usually not life-threatening, the pathogenesis and associated complications are not yet fully understood.

**Case description:**

A 54-year-old male was hospitalized due to a cough, accompanied by expectoration and dyspnea. After admission, the patient's breathing rapidly deteriorated, and despite the use of a ventilator, it was challenging to maintain respiratory function. While initiating extracorporeal membrane oxygenation (ECMO) and empirical anti-infection treatments, the alveolar lavage fluid was collected and examined by metagenomics next generation sequencing (mNGS). The mNGS result indicated *C. psittaci*. Subsequently, the anti-infection regimen was immediately adjusted. The respiratory function improved on the 13th day after admission, and ECMO was withdrawn. However, the patient developed abdominal distension and intestinal edema. After intravenous infusion therapy, intestinal ischemia and necrosis occurred and surgical resection was performed. The patient's condition improved after the operation and he was transferred to a local hospital for rehabilitation.

**Conclusion:**

This case report demonstrates the development of intestinal ischemic necrosis following severe *C. psittaci* pneumonia. This unique association has not been reported previously and highlights the importance of potential gastrointestinal complications in severe *C. psittaci* pneumonia, which are often underestimated. Timely diagnoses and treatments of such infections and complications are necessary to achieve favorable clinical outcomes.

## Introduction

The global prevalence of chlamydial infections in birds is alarmingly high. Cumulative evidence indicates that since 2012, the prevalence has remained relatively stable at around 20%. All avian species are potential sources of transmission for human psittacosis (1, 2). Transmission to humans occurs through inhalation of aerosolized dust from infected birds' feathers, dried feces, urine, other secretions, or through direct contact (3). *Chlamydophila psittaci* enters the bloodstream through the respiratory tract, primarily affecting the lungs. Infection symptoms include high fever, chills, headaches, sore throat, and muscle pain, often mimicking the flu. In severe cases, it can lead to respiratory failure and various complications, such as severe pneumonia (4). Beyond respiratory symptoms, *C. psittaci* multiplies in host cells as protozoa or reticulocytes, and replicates in mononuclear phagocytes in the liver and spleen, affecting not only the lungs but also other organs. However, the effects of *C. psittaci* on the digestive system are less well reported, especially in the gastrointestinal tract (2, 5, 6). Due to the non-specific clinical manifestations of *C. psittaci*, early identification is challenging. Among cases of community-acquired pneumonia, *C. psittaci* pneumoniae is relatively rare (< 1%), and the global clinical misdiagnosis rate of sporadic psittacosis is as high as 50%−80% (2, 7). With the widespread application of metagenomics next generation sequencing (mNGS), more cases of *C. psittaci* infection are being reported. Recent studies have documented the risk of interpersonal transmission of *C. psittaci*, which may be complicated by digestive system diseases such as pancreatitis, confirming that the pathogenesis of *C. psittaci* pneumonia and associated complications are not yet fully understood (6, 8, 9). This report presents a unique case of severe *C. psittaci* pneumonia, complicated by ischemic intestinal necrosis, a severe gastrointestinal complication that has not been previously reported or considered in the context of this infection.

## Case report

A 54-year-old male was admitted to the hospital after experiencing a cough and sputum production for 3 days, as well as fever and dyspnea for 1 day. He did not report any abdominal pain, diarrhea, or watery stools, and there were no indications of gastrointestinal ulcers. After admission, the highest body temperature was 40°C, heart rate: 131 bpm, blood pressure: 136/74 mmHg.

The β-D-glucan test and the galactomannan test were both negative. Respiratory pathogen spectrum IgM (Coxsackievirus B*, Mycoplasma pneumoniae*, parainfluenza virus, Influenza A virus, Influenza B virus*, Chlamydophila pneumoniae, Legionella pneumophila*, Coxsackievirus A, Echovirus, Respiratory syncytial virus, Adenovirus) were negative. Tests for Cytomegalovirus*, Toxoplasma*, Rubella virus, and Herpesvirus (IgG + IgM) were all negative, and the indicators for autoimmune diseases (anti-extractable nuclear antigen-antibody, anti-glomerular basement membrane antibody, anti-nuclear antibody and anti-neutrophilic cytoplasmic antibody) were all negative. On the same day, bronchoalveolar lavage fluid (BALF) was sent for mNGS analysis. Quantitative real-time Polymerase Chain Reaction performed for Influenza virus type A, influenza virus type B, respiratory syncytial virus, adenovirus, human rhinovirus, and coronavirus disease 2019 detection in his throat swab sample revealed a negative result.

**Figure 1:**
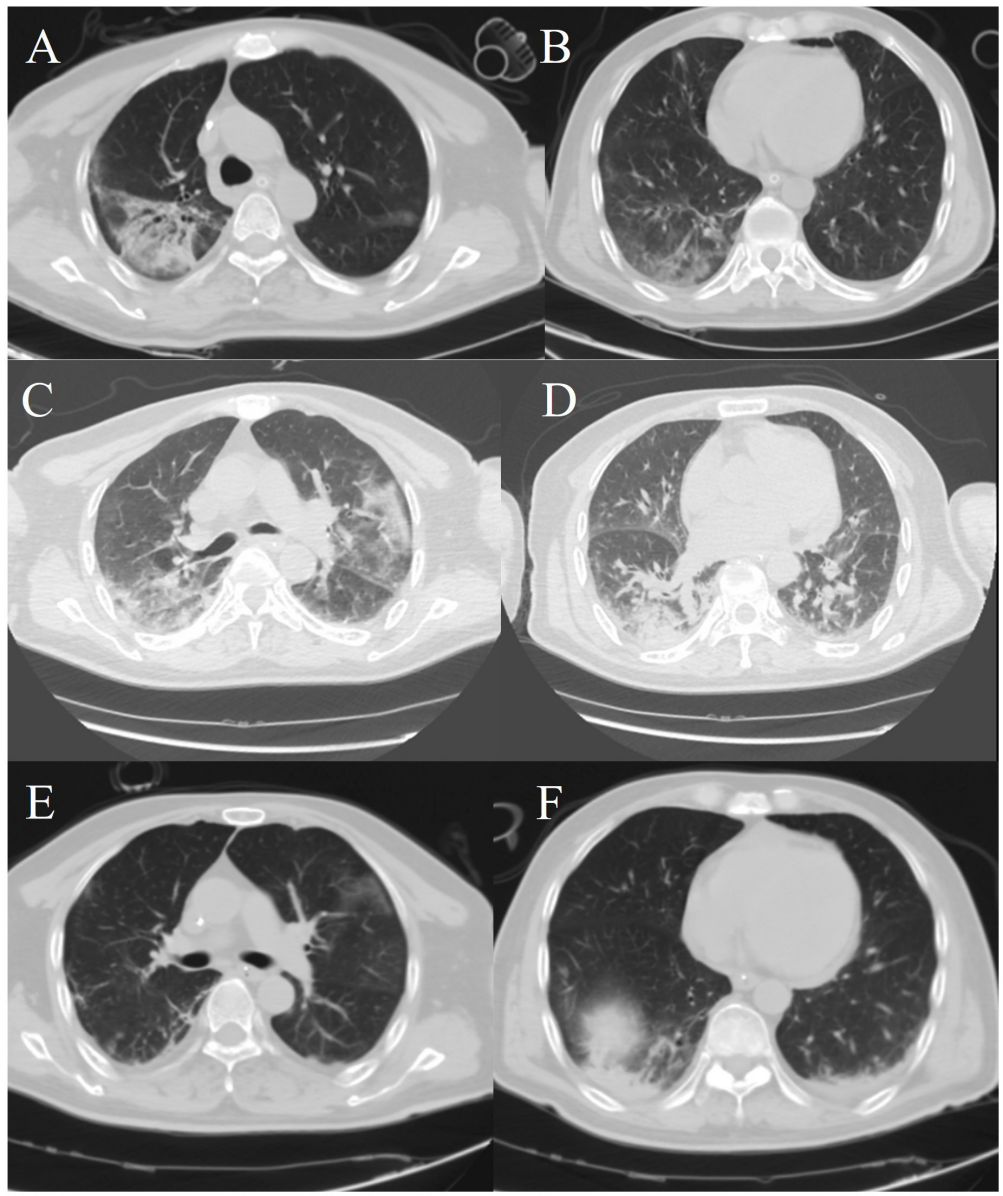
Chest computed tomography images. **(A, B)** The patient's chest CT scans on the first day after admission. **(C, D)** A chest computed tomography image was obtained on hospital day 13. **(E, F)** A chest computed tomography image was obtained on hospital day 23.

CT examination of the chest revealed: scattered patchy hyperdense and solid shadows in the upper lobe in both lungs, but predominantly in the right lung (Figures 1A, B). After admission, the patient's respiratory function progressively worsened, and it was difficult to maintain ventilation with non-invasive ventilator assistance. Consequently, endotracheal intubation, sedation and analgesia as well as anti-infection treatment with Imipenem-cilastatin and Tigecycline were carried out in early experimental applications. Ventilator parameters were FiO_2_: 100%, PEEP: 14 cmH_2_O and SpO_2_ was 78%. The oxygenation index was 42 mmHg. WBC: 21.53 × 10^9^/L, PCT: >100 ng/ml (normal < 0.25 ng/ml), IL-6: 1,646 ng/ml (normal ≤ 7 pg/ml), the CRP (concentration of C-reactive protein) was 351.9 mg/L. The albumin level was 25.1 g/L (normal 35–50 g/L). Alanine aminotransferase (ALT) was 34 U/L (normal 0–50 U/L), and aspartate aminotransferase (AST) was 101.2 U/L (normal 17–59 U/L). Bilirubin, creatinine, electrolytes, creatine kinase, creatine kinase isoenzyme cardiac troponin I, and concentrations of B-type natriuretic peptide were within normal limits. T-SPOT and Tuberculin tests were both negative. The total mature T Lymphocyte count in blood was 0.095 × 10^9^/L, PH: 7.36, Lac: 2.2 mmol/L, Sequential Organ Failure Assessment (SOFA) score: 12, Acute Physiology and Chronic Health Evaluation (APACHE) II score: 35, the risk of death was 84.86%. It was difficult to resolve the respiratory failure with ventilator support. So Veno-venous Extracorporeal Membrane Oxygenation (VV-ECMO, bilateral femoral vein, good function of ECMO after optimal positioning of blood collection end assessed by ultrasound) was performed, the patient was subjected to systemic administration of heparin for anticoagulation, along with symptomatic supportive treatment using proton pump inhibitors (PPIs) for acid suppression and gastroprotection.

**Figure 2:**
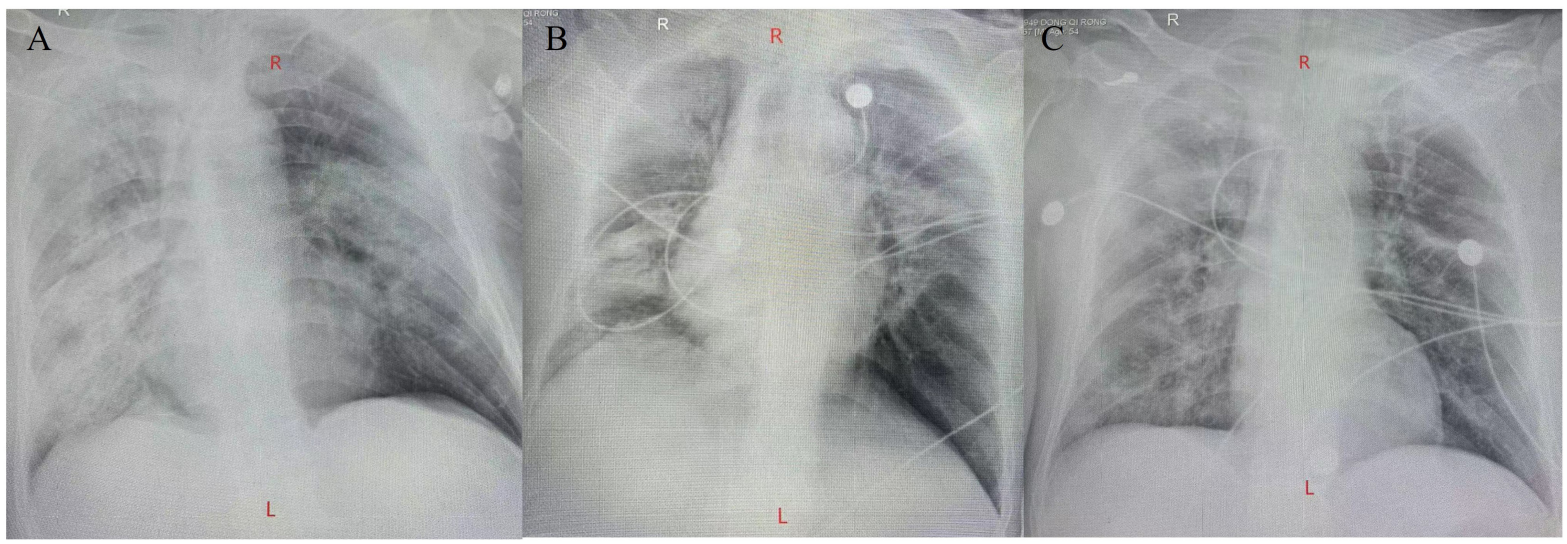
Chest X-ray imaging. **(A)** A chest X-ray imaging was obtained on hospital day 2. **(B)** A chest X-ray imaging was obtained on hospital day 8. **(C)** A chest X-ray imaging was obtained on hospital day 12.

Two days later, Chest X-ray imaging indicates inflammatory changes in both lungs (Figure 2A). The result of BALF mNGS showed that the causative agent of the lung infection was *C. psittaci*. The chlamydial genus exhibited a relative abundance of 98.5%, with a total of 142,586 sequences identified. Among these, 92,528 sequences corresponded to *C. psittaci*, confirmed with a confidence level of 99%. Additionally, testing for antibiotic-resistance genes returned negative results. Therefore, azithromycin was administered for anti-infection treatment. Meanwhile classical microbiological culture of blood and sputum for other typical bacteria yielded negative results.

**Figure 3:**
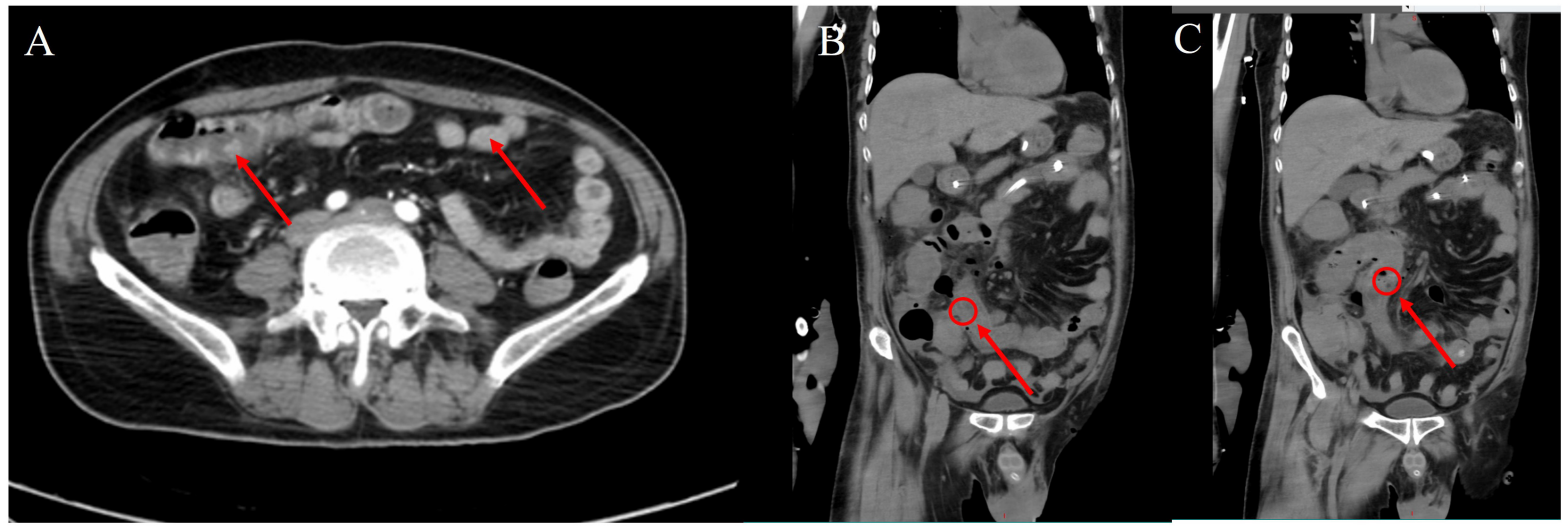
Abdominal computed tomography images. **(A)** On the 13th day after admission, the distal jejunal wall was thickened, edema was visible in the mesentery, the fat space was blurred, and the thickness of the tube wall was about 0.5 cm, and the contrast scan showed obvious enhancement. **(B, C)** On the 23rd day after admission, the intestinal wall is thickened and edematous, with a maximum thickness of about 0.9 cm, the density of the tube wall is increased, multiple free air shadows (suggesting perforation) can be seen in the adjacent fat space, and the mesangium and omentum are thickened.

**Figure 4:**
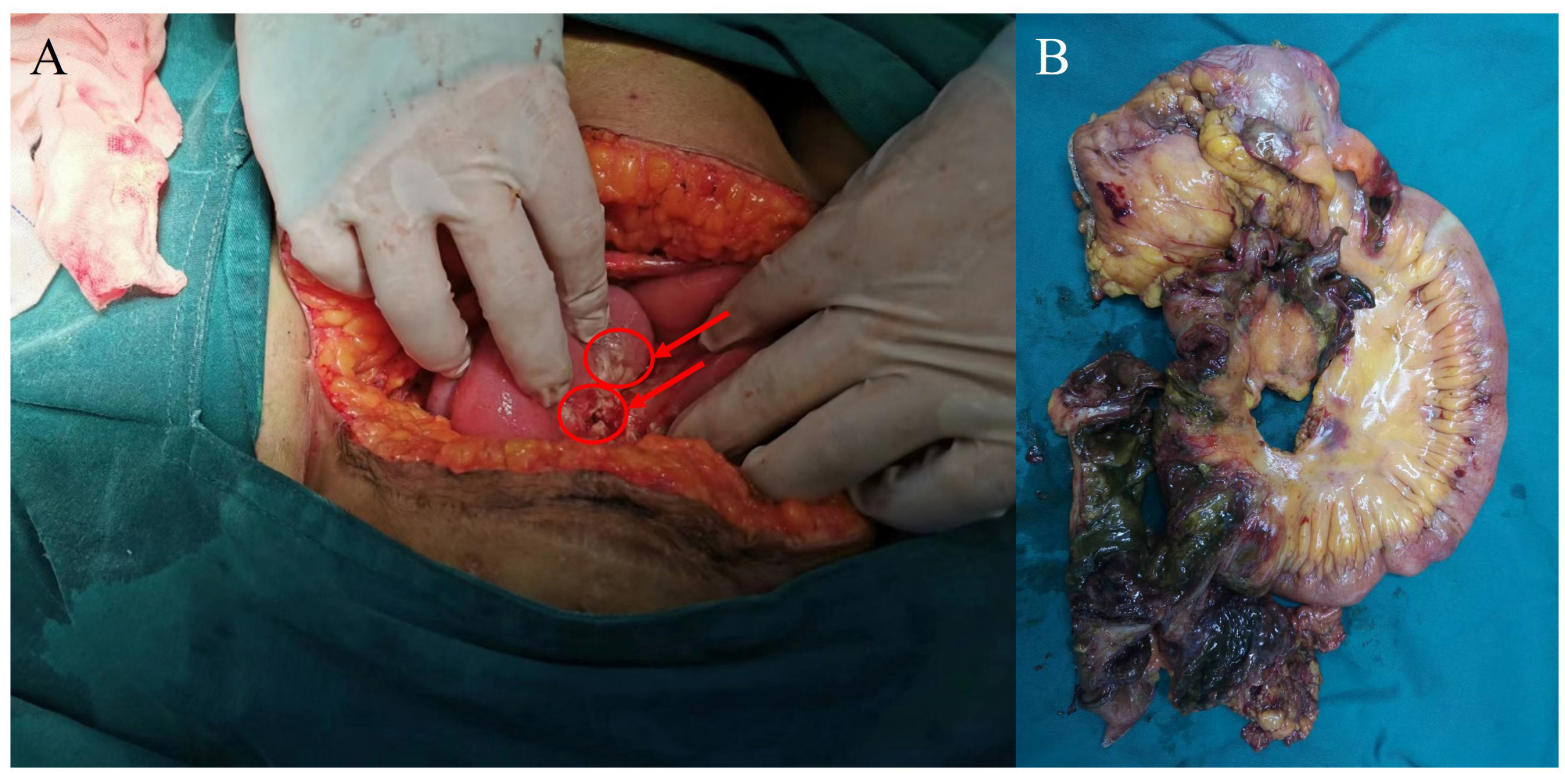
Photograph taken during surgery. **(A)** The right hemicolectomy (red arrowhead), surrounded by inflammatory thickened tissue. **(B)** Multiple perforations and necrosis of part of the ileum and right companion colon.

The patient's respiratory function gradually improved. The reexamination of the chest plain film showed a progressive improvement compared with the previous one (Figures 2B, C). He was finally successfully weaned off ECMO on the 13th day after admission. Chest CT examination indicated that there are inflammatory manifestations in both lower lobes of the lungs (Figures 1C, D). An abdominal CT revealed thickening of the distal small bowel wall, with evidence of mesenteric edema and blurred fat planes. The wall thickness was ~0.5 cm, with significant enhancement on the contrast-enhanced scan (CT value of 24 HU), lower than the normal bowel wall enhancement (Figure 3A). Enteral nutrition was temporarily stopped on the same day, while continuing treatment with PPIs. After 10 days of fluid therapy, a chest computed tomography image showed that the lung lesions are resolved (Figures 1E, F), and the patient experienced worsening abdominal distension, and another abdominal CT showed increased thickening and edema of the bowel wall, with the thickest portion measuring ~0.9 cm and increased wall density (CT value of 57.7 HU, suggestive bowel necrosis, Figures 3B, C). Multiple free gas shadows were seen in the adjacent fat planes (indicating perforation), as well as thickening of the mesentery and omentum. Emergency laparotomy revealed 6 perforations in the small intestine ~60 cm from the ileocecal junction, with large defects occupying about 1/2 of the intestinal circumference. Additionally, a 1 cm perforation was found in the ascending colon ~12 cm from the ileocecal junction. Postoperative pathology indicated extensive infiltration of inflammatory cells in the intestinal mucosa and submucosa. Disappointingly, the inclusion bodies of *Chlamydophila* was not observed in the intestinal mucosa. The diagnosis was confirmed as multiple ischemic necrosis and perforations of the ileum and ascending colon (Figure 4). Upon reexamination, multiple laboratory test indicators tended to be normal (Supplementary Figure 1).

The patient was discharged on the 5th day after surgery and underwent rehabilitation at a local hospital. At the 1-month follow-up after discharge, there were no neurological or limb function complications, and the patient was in a good psychological state.

## Discussion

*C. psittaci* is a Gram-negative pathogenic bacterium that primarily relies on the mononuclear phagocyte system for its growth and metabolism. This pathogenic *Chlamydophila* releases endotoxins, which prompt the body to initiate autoimmune and allergic responses. During the immune response to a chlamydial infection, T cells and infection-related cells exacerbate immune damage, ultimately leading to dysfunction in multiple organs (10, 11).

The incubation period for parrot fever, caused by *C. psittaci*, typically ranges from 5 to 14 days. *C. psittaci* can infect various tissues or organs. The severity of the disease varies from asymptomatic infections to fulminant sepsis. Clinical symptoms depend on the severity of the illness, the organs affected, and the presence of any comorbidities or complications. In cases involving gastrointestinal damage, patients with psittacosis often exhibit significant liver involvement, characterized by elevated transaminase levels and hepatosplenomegaly. Some patients may initially present with gastrointestinal symptoms, which primarily include discomfort like nausea, vomiting, abdominal pain, and diarrhea. Severe cases may reveal additional signs, including jaundice and melena (4).

Intestinal ischemia refers to a series of disorders that occur due to a slowdown or cessation of blood flow, which may cause pain. If not treated promptly, this condition may advance to severe intestinal necrosis, which is life-threatening. Its incidence is quite low, estimated at about 0.09%−0.2% (12). High-risk factors for the occurrence of intestinal ischemic necrosis include: age over 50 years, atherosclerosis, volvulus, tumors, a long history of smoking, cardiovascular diseases such as atrial fibrillation, drugs that cause vasoconstriction such as contraceptives, a hypercoagulable state, hypertension, diabetes, the use of cocaine and methamphetamine, etc. (13). Seeking medical attention promptly can significantly improve recovery outcomes. Secondary intestinal ischemic necrosis resulting from psittacosis has not been recorded previously, likely due to the low incidence of psittacosis itself. While there have been previous reports of abdominal pain and hematemesis linked to *C. psittaci*, most research has focused on its effects on the pulmonary inflammatory response. The rapid progression of *C. psittaci* infection may be associated with excessive activation and continual release of inflammatory factors. However, there has been relatively little focus on understanding the causes and mechanisms behind the abdominal symptoms that can arise from *C. psittaci* infection (14).

From mild influenza-like symptoms to systemic infections, the primary manifestation of *C. psittaci* is atypical pneumonia. Common symptoms of *C. psittaci* pneumonia are high fever, cough, and dyspnea. Gastrointestinal symptoms such as anorexia and diarrhea can ensue due to high fever (10, 15). With the quarantine of imported birds and improved veterinary hygiene measures, outbreaks of *C. psittaci* are now rare, with only a few disseminated cases, and even fewer manifestations of severe pneumonia that require intensive care, while endocarditis, myocarditis, and neurologic complications have been reported in patients with severe pneumonia (3, 13, 16).

In our case, most classical microbiological tests returned negative results. Initially, we considered the possibility of atypical pathogen infections. But other viral infections could not be ruled out. Given the low detection rate of routine laboratory methods for atypical pathogens and the patient's critical condition, we needed swift microbiological evidence. Therefore, we submitted BALF for mNGS. *C. psittaci* is not typically included in traditional microbiological diagnostics. While culturing for psittacosis aids in diagnosis and is highly reliable, it has a low detection rate and is time-consuming, which hampers rapid clinical diagnosis. The clinical misdiagnosis rate for *C. psittaci* is high, often leading to underestimation and insufficient diagnosis. The use of mNGS has increased the number of patients diagnosed with *C. psittaci* pneumonia caused by *C. psittaci*. We deemed the results from mNGS to be reliable and did not submit additional hematological or serological tests.

The multidisciplinary health care team excluded other common causes of intestinal diseases (such as *Clostridioides difficile* infection or organic diseases of the small intestine and colon). We timely and effectively implemented advanced life support methods to prevent the rapid progression of the disease and maintain the stability of various organ functions. Looking back on the entire treatment process, there were no obvious hemodynamic disturbances, no high doses of vasoactive drugs, and no factors affecting the intestinal blood supply due to the VV-ECMO catheter position. Although there have been some reports of VV-ECMO causing gastrointestinal bleeding in the past, these are believed to be caused by perforation of stress ulcers in the gastrointestinal tract during treatment (17). In our search for alternative causes, we thoroughly reviewed the patient's entire disease course. The patient did not present with prolonged hypotension or shock, had no history of gastrointestinal disease. Abdominal vascular screening was conducted at the onset of ECMO, but we did not find any significant issues. Unfortunately, intestinal ischemic necrosis occurred late in the overall progression of the disease. By that time, we had already administered anti-infection therapy, so even if there had been inclusion bodies in the lesion, they would have been difficult to find at this time. Although the mechanism by which *C. psittaci* infection might cause intestinal necrosis remains unclear, we speculate that it may be due to an excessive immune response triggered by the infection, which negatively impacts intestinal tissues and leads to necrosis (18, 19). After extensive discussions, both the intensive care physician and the cardiac surgeon concluded that the intestinal ischemic necrosis observed in this case was most likely related to *C. psittaci* infection. Although severe pneumonia was the primary clinical symptom, the pathogen also impacted other organs, including the gastrointestinal tract and triggered an inflammatory reaction. The necrosis and perforation of the lower digestive tract may be related to the previous severe *C. psittaci* infection in the gastrointestinal tract. We promptly discontinued enteral nutrition upon discovering abnormal intestinal symptoms, the patient's condition was severe, and in conjunction with “The Sanford Guide To Antimicrobial Therapy 2018,” azithromycin was administered intravenously to combat infection (20). In the mouse model of *C. psittaci* pneumonia, treatment with azithromycin for 7 days lead to a survival rate of 100% (21). Furthermore, decades of cumulative research on the lytic properties of azithromycin have established it in as a safe, short-term treatment for various diseases, including chlamydiosis. This is mainly due to its ability to modulate multiple immune subsystems in response to different infections, all while maintaining physiological homeostasis (22). However, it cannot be excluded that enteral nutrition and azithromycin administered during treatment may also have accelerated disease progression (23). The main course of intestinal ischemic necrosis occurred in the 10 days after ECMO withdrawal, during which the patient received only supportive anti-infectious treatment and rehabilitation exercises. Previously reported complications such as gastrointestinal perforation are often related to irregular prevention of gastrointestinal ulcers during treatment or underlying causes such as intestinal inflammation, stress and high doses of steroids (24).

In our case, although there is no direct evidence linking intestinal necrosis to *C. psittaci*, perforation and necrosis in the small intestine and colon occurred in the late stage of severe *C. psittaci* pneumonia while the patient was receiving advanced life support including ECMO and post-weaning treatment. His gastrointestinal tract was adequately protected. *C. psittaci* may be a potential culprit for lower gastrointestinal ischemic necrosis.

## Conclusion

This case report highlights the risk of lower gastrointestinal ischemic necrosis and perforation associated with severe *C. psittaci* pneumonia, even in the later stages of the illness. However, the exact mechanism by which *C. psittaci* leads to intestinal necrosis requires further investigation. For patients with rapidly progressing severe *C. psittaci* pneumonia, while advanced life support can stabilize vital signs, prompt and accurate diagnosis along with targeted anti-infection treatment is crucial for a favorable prognosis.

## Data Availability

The original contributions presented in the study are included in the article/Supplementary material, further inquiries can be directed to the corresponding author.
