# Supplementary material for: Chlamydophila psittaci pneumonia followed by lower gastrointestinal ischemic necrosis: a case report
Source: Front Med (Lausanne). 2025 Jan 8;11:1394897. doi: 10.3389/fmed.2024.1394897 (PMC11751233; doi:10.3389/fmed.2024.1394897)
Supplement: Supplementary file 1 [file Supplementary_file_1.docx]

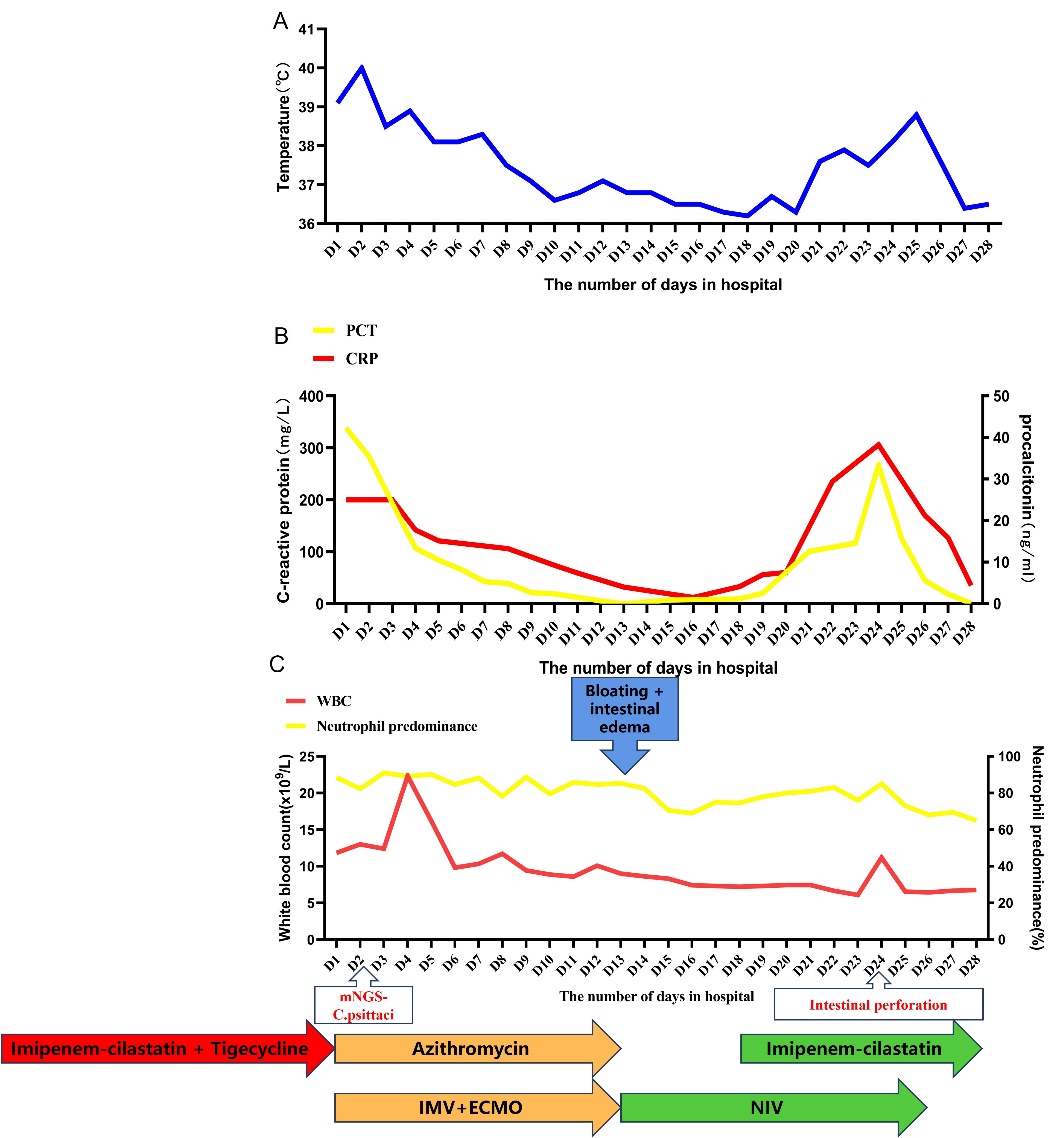


**Supplementary Fig.1.**Results of laboratory tests of the patient at different times. (A)Change in Body temperature during hospitalization. (B)Change in C-reactive protein and procalcitonin during hospitalization. (C)white blood cell count, neutrophil predominance and antimicrobial treatment during hospitalization
